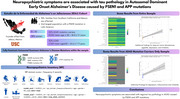# Neuropsychiatric symptoms are associated with tau pathology in Autosomal Dominant Early Onset Alzheimer's Disease caused by *PSEN1* and *APP* mutations

**DOI:** 10.1002/alz70857_105799

**Published:** 2025-12-25

**Authors:** Nancy E Ortega, Judy Pa, Yonggand Shi, Jiaxin Yue, Abhay P Sagare, Helena C Chui, John M Ringman

**Affiliations:** ^1^ Alzheimer's Disease Cooperative Study (ADCS), University of California San Diego, La Jolla, CA, USA; ^2^ Neurosciences Graduate Program, University of California San Diego, La Jolla, CA, USA; ^3^ Alzheimer's Disease Cooperative Study (ADCS), University of California, San Diego, La Jolla, CA, USA; ^4^ Neuroscience Graduate Program, University of California San Diego, La Jolla, CA, USA; ^5^ Department of Neurology, Stevens Neuroimaging and Informatics Institute, Keck School of Medicine, University of Southern California, Los Angeles, CA, USA; ^6^ Stevens Neuroimaging and Informatics Institute, Keck School of Medicine, University of Southern California, Los Angeles, CA, USA; ^7^ Zilkha Neurogenetic Institute, Keck School of Medicine, University of Southern California, Los Angeles, CA, USA; ^8^ Alzheimer's Disease Research Center, Keck School of Medicine, University of Southern California, Los Angeles, CA, USA; ^9^ Department of Neurology, Keck School of Medicine, University of Southern California, Los Angeles, CA, USA; ^10^ Keck School of Medicine, University of Southern California, Los Angeles, CA, USA

## Abstract

**Background:**

This study aimed to characterize the relationship between neuropsychiatric symptoms and tau pathology in vivo within a cohort of participants of predominantly Mexican ancestry at risk for fully penetrant Autosomal Dominant Early Onset Alzheimer's disease (ADAD) caused by mutations in PSEN1 and APP genes.

**Method:**

Participants from the Estudio de la Enfermedad de Alzheimer's en Jaliscienses (EEAJ) cohort were included based on availability of demographic variables, genetic mutation carrier status, tau PET scans (flortaucipir), and completion of the Neuropsychiatric Inventory (NPI). This sample included participants with or known to be at‐risk for a mutation within the PSEN1 or APP genes, as well as individuals with sporadic late‐onset Alzheimer's disease (LOAD). Linear regression models were used to examine the relationship between individual NPI domain frequency x severity scores and regional tau PET standard uptake value ratios (SUVrs). Associations within each hemisphere were independently investigated to examine lateralization effects. All twelve NPI domains were evaluated. Additional analyses were conducted only on ADAD mutation carriers. Models were adjusted for age, gender, ADAD versus LOAD, mutation‐specific estimated years to dementia diagnosis, and mutation carrier status. All results were deemed significant at *p* <0.05. The Benjamini–Hochberg procedure was used to control for false discovery rate.

**Result:**

The sample included 56 participants (59% women, 32% LOAD, 52% ADAD mutation carriers [83% PSEN1 A431E, 10% PSEN1 I180F, 3% PSEN1 F338S, and 3% APP V717I carriers], EOAD mean age ± SD = 36.8±11.1 years, LOAD mean age±SD = 65.6±7.71 years). Analyses evaluating the entire sample found aberrant motor disturbances were associated with tau pathology within the entorhinal cortex and temporal pole. Apathy was found to have a significant relationship with tau pathology within the left posterior cingulate cortex. Tau pathology within the left temporal pole was associated with appetite. Depression was associated with the superior frontal gyrus and the left posterior cingulate cortex. For irritability there were significant associations within the left hemisphere for the entorhinal cortex and the right frontal pole.

**Conclusion:**

Neuropsychiatric symptoms are associated with regional tau pathology in ADAD caused by PSEN1 and APP mutations.